# Trophic Relationships and Habitat Preferences of Delphinids from the Southeastern Brazilian Coast Determined by Carbon and Nitrogen Stable Isotope Composition

**DOI:** 10.1371/journal.pone.0082205

**Published:** 2013-12-16

**Authors:** Tatiana Lemos Bisi, Paulo Renato Dorneles, José Lailson-Brito, Gilles Lepoint, Alexandre de Freitas Azevedo, Leonardo Flach, Olaf Malm, Krishna Das

**Affiliations:** 1 Laboratório de Mamíferos Aquáticos e Bioindicadores “Profa. Izabel Gurgel” (MAQUA), Faculdade de Oceanografia, Universidade do Estado do Rio de Janeiro (UERJ), Rio de Janeiro, RJ, Brazil; 2 Programa de Pós-Graduação em Ecologia, Universidade Federal do Rio de Janeiro (UFRJ), Rio de Janeiro, RJ, Brazil; 3 Laboratório de Radioisótopos Eduardo Penna Franca, Instituto de Biofísica Carlos Chagas Filho, Universidade Federal do Rio de Janeiro (UFRJ), Rio de Janeiro, RJ, Brazil; 4 Laboratoire d'Oceanologie - MARE, Université de Liège, Liège, Belgique; 5 Instituto Boto-cinza, Mangaratiba, RJ, Brazil; University of Connecticut, United States of America

## Abstract

To investigate the foraging habitats of delphinids in southeastern Brazil, we analyzed stable carbon (δ^13^C) and nitrogen (δ^15^N) isotopes in muscle samples of the following 10 delphinid species: *Sotalia guianensis*, *Stenella frontalis, Tursiops truncatus, Steno bredanensis, Pseudorca crassidens*, *Delphinus* sp., *Lagenodelphis hosei, Stenella attenuata, Stenella longirostris* and *Grampus griseus*. We also compared the δ^13^C and δ^15^N values among four populations of *S. guianensis*. Variation in carbon isotope results from coast to ocean indicated that there was a significant decrease in δ^13^C values from estuarine dolphins to oceanic species. *S. guianensis* from Guanabara Bay had the highest mean δ^13^C value, while oceanic species showed significantly lower δ^13^C values. The highest δ^15^N values were observed for *P. crassidens* and *T. truncatus*, suggesting that these species occupy the highest trophic position among the delphinids studied here. The oceanic species *S. attenuata*, *G. griseus* and *L. hosei* had the lowest δ^15^N values. Stable isotope analysis showed that the three populations of *S. guianensis* in coastal bays had different δ^13^C values, but similar δ^15^N results. Guiana dolphins from Sepetiba and Ilha Grande bays had different foraging habitat, with specimens from Ilha Grande showing more negative δ^13^C values. This study provides further information on the feeding ecology of delphinids occurring in southeastern Brazil, with evidence of distinctive foraging habitats and the occupation of different ecological niches by these species in the study area.

## Introduction

Delphinidae constitutes the richest taxonomical family of all cetaceans, with 36 currently recognized species. The presence of delphinids along the Rio de Janeiro coast has been reported from direct observation or from stranding records [1], [2], [3], [4]. These species are distributed within bays and estuaries (e.g., Guiana dolphin, *Sotalia guianensis*), as well as along the continental shelf and in oceanic environments off the coast of Rio de Janeiro State. However, there is little information regarding the habitat preferences and feeding ecology of delphinids from the study area. Most of the species, including false killer whale (*Pseudorca crassidens*), Risso's dolphin (*Grampus griseus*), spinner dolphin (*Stenella longirostris*), Fraser's dolphin (*Lagenodelphis hosei*) and pantropical spotted dolphin (*Stenella attenuata*), have been observed opportunistically because they usually occupy off-shore areas. Investigating the habitat preferences and the trophic relationships among the delphinid species is of great importance for understanding the roles and ecological niches occupied by these animals in marine food webs. This information will make it possible to better understand the degree of overlap and segregation of delphinids in the foraging area in southeastern Brazil.

Rio de Janeiro State is located along the southeastern Brazilian coast. This region is under high anthropogenic pressure because it is an important urban and industrial center for Brazil [5], [6], [7], [8]. Harbor activities, oil refineries, oil and natural gas exploration, seismic prospecting, expanding industrial parks, intense vessel traffic and intense commercial fishing are also important sources of impact along the Rio de Janeiro coast [5], [9], [10]. In the face of this anthropogenic pressure, ecological research on delphinids, including on such topics as trophic relationships and habitat preferences, is required to assess and monitor the potential threats to these animals in marine environments [11]. For most of the delphinid species in southeastern Brazil, basic ecological knowledge is still scarce.

The more traditional methods used for studying the feeding ecology of cetaceans relies on stomach content analyses from stranded or accidentally caught animals [12], [13], [14]. This approach makes it possible to identify the species consumed; however, the technique used fragments of preys in different stages of digestion, which can lead to over- or underestimation of the importance of certain prey species and consequently hinder the interpretation of dolphin feeding habits [15], [16]. In addition, the use of stranded animals can be biased, reflecting the diet of sick or injured animals that were not feeding normally before dying [17], [18].

The analysis of carbon and nitrogen stable isotopes has been shown to be a useful complementary tool for investigating foraging and feeding behavior of cetaceans [19], [20], [21]. The usefulness of the technique is a consequence of the fact that the stable-isotope composition of predators reflects prey signatures assimilated over time [22], [23]. Carbon isotope (δ^13^C) values have been used to trace the primary source of carbon in the food web because this isotope is indicative of low trophic enrichment (1–2‰) [24], [25]. Thus, it is possible to differentiate food sources originating from the following systems: terrestrial versus marine, coastal versus oceanic, or benthic versus pelagic [24], [26], [27]. In addition, δ^13^C values of particulate organic matter (POM) and phytoplankton can vary along a gradient of coastal to oceanic regions, with higher δ^13^C values in waters closer to the coast [28]. Thus, it is possible to investigate the foraging area and geographical variation in the use of the region by cetaceans, as well as to differentiate coastal species or populations from oceanic ones [19], [21], [29]. Nitrogen isotopes (δ^15^N) have been used to study trophic relationships in marine food webs and to assess trophic levels [20], [30]. This is possible due to the relationship between δ^15^N values and the trophic position that an organism occupies [31], [32].

Stable carbon and nitrogen isotope analyses were performed using delphinid muscle to 1) investigate the foraging area and trophic relationships of 10 delphinid species from southeastern Brazil, 2) compare the stable isotope values among four Guiana dolphin populations from the coast of Rio de Janeiro State, and 3) identify possible trophic differences between sexes and among age classes of Guiana dolphins.

## Materials and Methods

### Ethics Statement

Muscle samples of delphinids were collected with appropriate permissions from Brazilian Environmental Agencies – IBAMA/MMA (permission number 11495-1) and ICMBio/MMA (permission number 11579-1).

### Sampling

**Figure 1:**
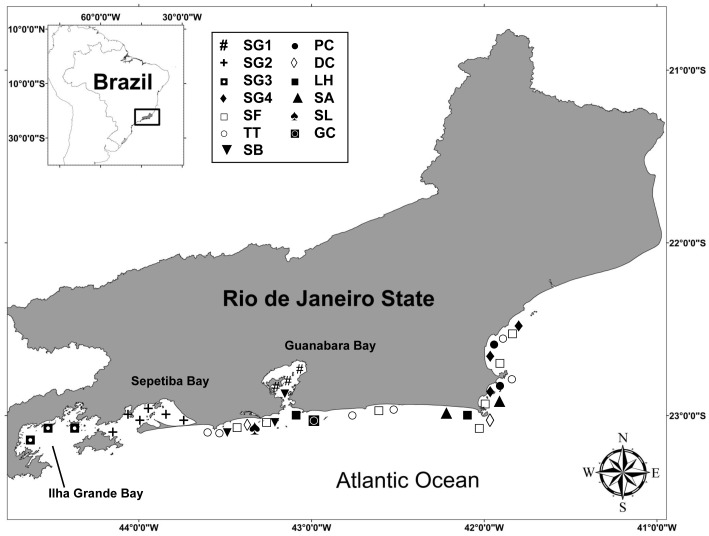
Map of the study area in Rio de Janeiro State, southeastern Brazil. Stranding sites of delphinids are shown. SG1 - *Sotalia guianensis* from Guanabara Bay, SG2 - *S. guianensis* from Sepetiba Bay, SG3 - *S. guianensis* from Ilha Grande Bay, SG4 - *S. guianensis* from “Região dos Lagos”, SB - *Steno bredanensis*, TT - *Tursiops truncatus*, SF - *Stenella frontalis*, PC - *Pseudorca crassidens*, DC - *Delphinus* sp., LH - *Lagenodelphis hosei*, SA - *Stenella attenuata*, SL - *Stenella longirostris* and GG - *Grampus griseus*.

Muscle samples of 10 delphinids species (131 individuals) were obtained from specimens either incidentally caught in gillnet fisheries or stranded on the beaches of Rio de Janeiro State in southeastern Brazil from 1994 to 2009 (Fig. 1). The following species were targeted: Atlantic spotted dolphin, *Stenella frontalis* (n = 13), bottlenose dolphin, *Tursiops truncatus* (n = 7), rough-toothed dolphin, *Steno bredanensis* (n = 3), false killer whale, *Pseudorca crassidens* (n = 2), common dolphin, *Delphinus* sp. (n = 2), Fraser's dolphin, *Lagenodelphis hosei* (n = 10), pantropical spotted dolphin, *Stenella attenuata* (n = 2), spinner dolphin, *Stenella longirostris* (n = 1), and Risso's dolphin, *Grampus griseus* (n = 1), and 4 populations of Guiana dolphin, *Sotalia guianensis*, from Guanabara Bay (n = 26), Sepetiba Bay (n = 49), Ilha Grande Bay (n = 10) and “Região dos Lagos” (n = 5).

### Analysis of δ^13^C and δ^15^N

Muscle samples were dried at 60°C for 72 h and then ground into a homogeneous powder. Dried samples (∼1.5 mg) were weighed and placed in tin capsules (3×5 mm), and carbon and nitrogen stable isotope measurements were performed on a V.G. Optima (Isoprime UK) isotope ratio mass spectrometer coupled to an N-C-S elemental analyzer (Carlo Erba). Stable isotope ratios were expressed in delta notation as parts per thousand according to the following equation: 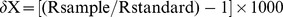
where X is ^13^C or ^15^N and R is the corresponding ratio of ^13^C/^12^C or ^15^N/^14^N. Carbon and nitrogen ratios were expressed in relationship to the V-PDB (Vienna Peedee Belemnite) standard and to atmospheric nitrogen, respectively. Reference materials (IAEA CH-6 and IAEA-N1) were also analyzed. The standard deviation on replicated measurements from a single delphinid sample was ±0.3‰.

Because lipids have been shown to be depleted in ^13^C and lipid tissue content can be variable [24], we measured the elemental content and calculated the sample C∶N ratio to verify the lipid content of each sample [33]. A total of 24 samples presented C∶N>3.5; therefore, we normalization the δ^13^C values according to the following equation [33]: 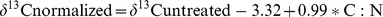



### Statistical analysis

The Kolmogorov-Smirnov test was used to test for normality of the data (K-S d = 0.083 and d = 0.081, p>0.20). Analyses of variance (ANOVA), followed by an Unequal N HSD *post-hoc* test, were used to compare carbon and nitrogen isotope values among species; dolphin calves were excluded from these analyses. In addition, we performed a cluster analysis aiming to detect isotopic patterns among delphinids species. For this analysis, we used Ward's method (minimum variance) and Euclidean distances [34]. ANOVAs were also used to verify differences in δ^13^C and δ^15^N values among adult males, adult females and calves of Guiana dolphins from Guanabara and Sepetiba bays. The Student's *t*-test was performed to compare male and female dolphins from Ilha Grande Bay.

## Results

**Figure 2:**
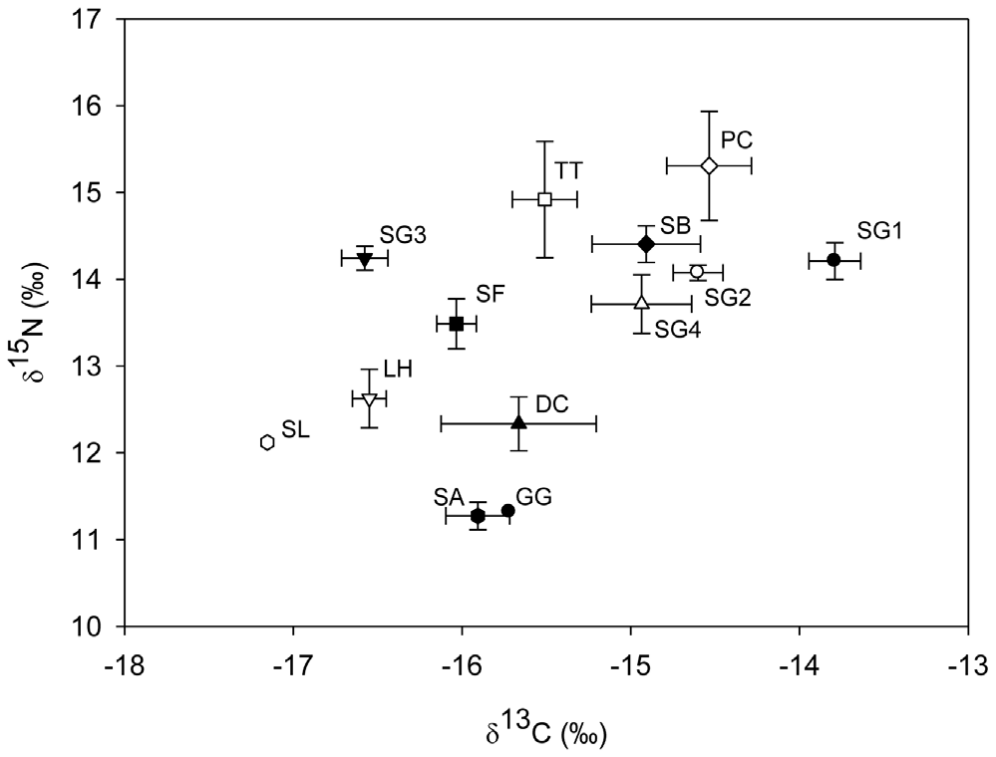
Mean (±SE) δ^13^C and δ^15^N values for delphinid muscle tissues from specimens collected from Rio de Janeiro State. • *Sotalia guianensis* from Guanabara Bay (SG1), ○ *S. guianensis* from Sepetiba Bay (SG2), ▾ *S. guianensis* from Ilha Grande Bay (SG3), ▵ *S. guianensis* from “Região dos Lagos” (SG4), ♦ *Steno bredanensis* (SB), □ *Tursiops truncatus* (TT), ▪ *Stenella frontalis* (SF), ⋄ *Pseudorca crassidens* (PC), ▴ *Delphinus* sp. (DC), ▿ *Lagenodelphis hosei* (LH), *Stenella attenuata* (SA), *Stenella longirostris* (SL) and • *Grampus griseus* (GG).

For the analyses, the four populations of Guiana dolphins occurring along the Rio de Janeiro State coast were treated as distinct groups. Three of these populations use inner areas of coastal bays (i.e., Guanabara Bay, Ilha Grande Bay and Sepetiba Bay) and the fourth occurs along the coast in an area known as “Região dos Lagos”. Mean δ^13^C and δ^15^N values from the 10 delphinids species from the Rio de Janeiro State coast ranged from −17.1 to −13.8‰ and from 11.3 to 15.3‰, respectively (Table 1, Fig. 2). These values varied significantly among species (ANOVA, δ^13^C: F_(10,107)_ = 18.64, p<0.0001 and δ^15^N: F_(10, 107)_ = 7.04, p<0.0001) (Table 2). Statistical tests could not be performed using data from spinner and Risso's dolphins (n = 1).

**Table 1:** Mean (±SD) δ^13^C and δ^15^N values in delphinids muscle tissues from the coast of Rio de Janeiro State, southeastern Brazil.

Common name	Species	n	δ^13^C (‰)		δ^15^N (‰)	
			mean ±SD	min/max	mean ±SD	min/max
Guiana dolphin	*Sotalia guianensis*					
	Guanabara Bay	20	−13.8±0.7	−15.3/−12.5	14.2±0.9	12.2/16.2
	Sepetiba Bay	44	−14.6±0.9	−16.9/−12.8	14.1±0.6	12.9/15.5
	Ilha Grande Bay	10	−16.6±0.4	−17.3/−16.0	14.2±0.2	13.5/15.3
Guiana dolphin	*Sotalia guianensis*					
	“Região dos Lagos”	5	−14.9±0.6	−15.7/−13.9	13.7±0.7	12.6/14.5
Atlantic spotted dolphin	*Stenella frontalis*	13	−16.0±0.4	−16.6/−15.4	13.5±1.0	12.2/15.4
Bottlenose dolphin	*Tursiops truncatus*	7	−15.5±0.5	−16.1/−14.6	14.9±1.7	11.6/16.7
Rough-toothed dolphin	*Steno bredanensis*	3	−14.9±0.5	−15.4/−14.3	14.4±0.3	14.1/14.8
False killer whale	*Pseudorca crassidens*	2	−14.5±0.3	−14.8/−14.3	15.3±0.9	14.7/15.9
Common dolphin	*Delphinus* sp.	2	−15.6±0.6	−16.1/−15.2	12.3±0.4	12.0/12.6
Fraser's dolphin	*Lagenodelphis hosei*	10	−16.5±0.3	−17.2/−16.0	12.6±1.0	10.0/13.8
Pantropical spotted dolphin	*Stenella attenuata*	2	−15.8±0.2	−16.1–15.7	11.4±0.2	11.1/11.4
Spinner dolphin	*Stenella longirostris*	1	−17.1	-	12.1	-
Risso's dolphin	*Grampus griseus*	1	−15.7	-	11.3	-

**Table 2:** Results of the Unequal N HSD *post-hoc* test for multiple comparisons of δ^13^C (upper-right) and δ^15^N (lower-left) values from samples of delphinid muscle tissues collected from the coast of Rio de Janeiro State, southeastern Brazil.

	SG1	SG2	SG3	SG4	SF	TT	SB	PC	DC	LH	SA
SG1		**0.03**	**0.00**	0.35	**0.00**	**0.00**	0.75	0.99	0.30	**0.00**	0.15
SG2	0.99		**0.00**	0.99	**0.00**	0.45	0.99	1.00	0.93	**0.00**	0.80
SG3	1.00	0.99		**0.02**	0.86	0.22	0.19	0.19	0.97	1.00	0.99
SG4	0.99	0.99	0.99		0.42	0.97	1.00	0.99	0.99	**0.03**	0.96
SF	0.57	0.82	0.69	0.99		0.96	0.74	0.64	0.99	0.89	1.00
TT	0.91	0.77	0.93	0.52	0.09		0.99	0.96	1.00	0.25	0.99
SB	1.00	0.99	1.00	0.99	0.96	0.99		0.99	0.99	0.21	0.95
PC	0.97	0.94	0.97	0.76	0.59	0.99	0.99		0.91	0.21	0.75
DC	0.54	0.65	0.52	0.88	0.96	0.11	0.39	**0.03**		0.98	1.00
LH	**0.00**	**0.01**	**0.00**	0.66	0.50	**0.00**	0.31	0.09	1.00		0.99
SA	**0.04**	0.06	**0.03**	0.17	0.29	**0.00**	**0.02**	**0.00**	0.97	0.09	

**Figure 3:**
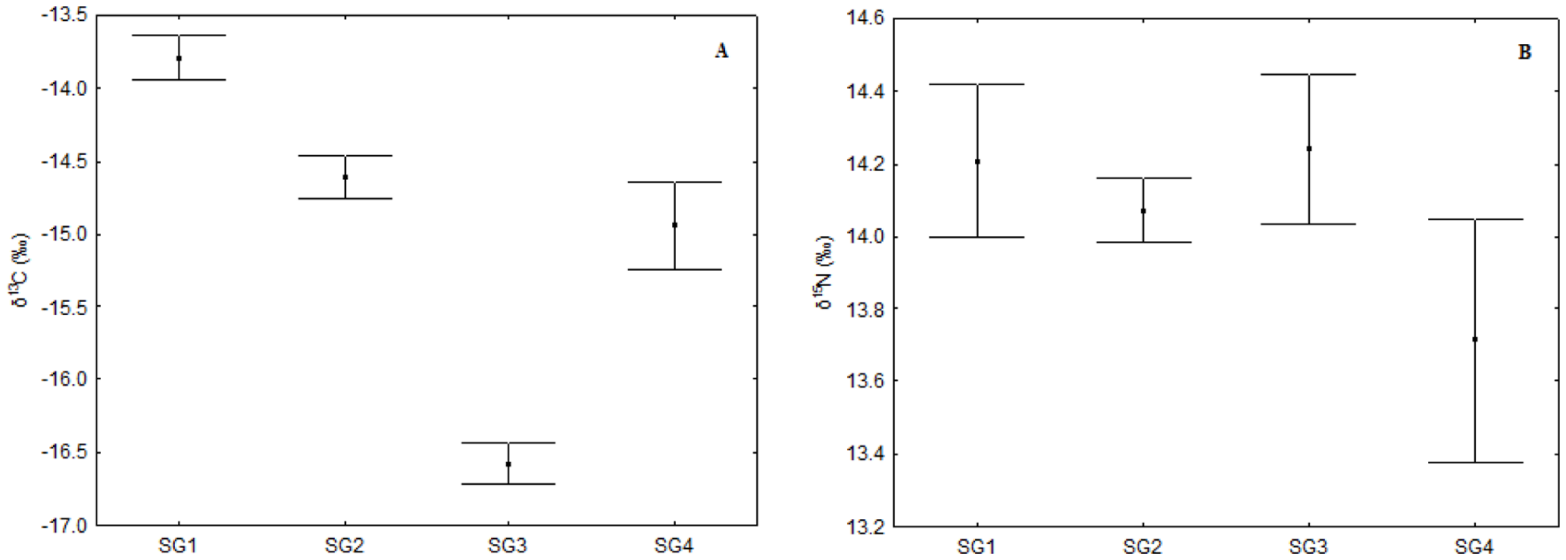
Mean (±SE) δ^13^C and δ^15^N values for Guiana dolphin muscle tissues. Specimens are from Guanabara Bay (SG1), Sepetiba Bay (SG2), Ilha Grande Bay (SG3) and “Região dos Lagos” (SG4), Rio de Janeiro State, southeastern Brazil. (A) δ^13^C values; (B) δ^15^N values.

Of the four populations of Guiana dolphins, the specimens from Guanabara Bay exhibited the highest δ^13^C values, while dolphins from Sepetiba Bay and “Região dos Lagos” had intermediate values and individuals from the Ilha Grande Bay had the lowest δ^13^C values (Unequal N HSD test; p<0.03) (Table 2). However, there was no difference in δ^15^N values among these populations (Unequal N HSD test; p>0.99) (Fig. 3).

Guiana dolphins from Guanabara Bay also displayed significantly higher δ^13^C values than Atlantic spotted, bottlenose and Fraser's dolphins (Unequal N HSD test, p<0.05; Table 2). False killer whales and bottlenose dolphins had higher δ^15^N mean values compared to common, Fraser's and pantropical spotted dolphins (Unequal N HSD test, p<0.05; Table 2). The lowest δ^13^C and δ^15^N values were observed for oceanic delphinids (i.e., spinner, Risso's, Fraser's and pantropical spotted dolphins). We found significant differences between the oceanic species (i.e., Fraser's and pantropical spotted dolphins) and Guiana dolphins for both δ^13^C and δ^15^N values (Unequal N HSD test, p<0.05; Table 2).

**Figure 4:**
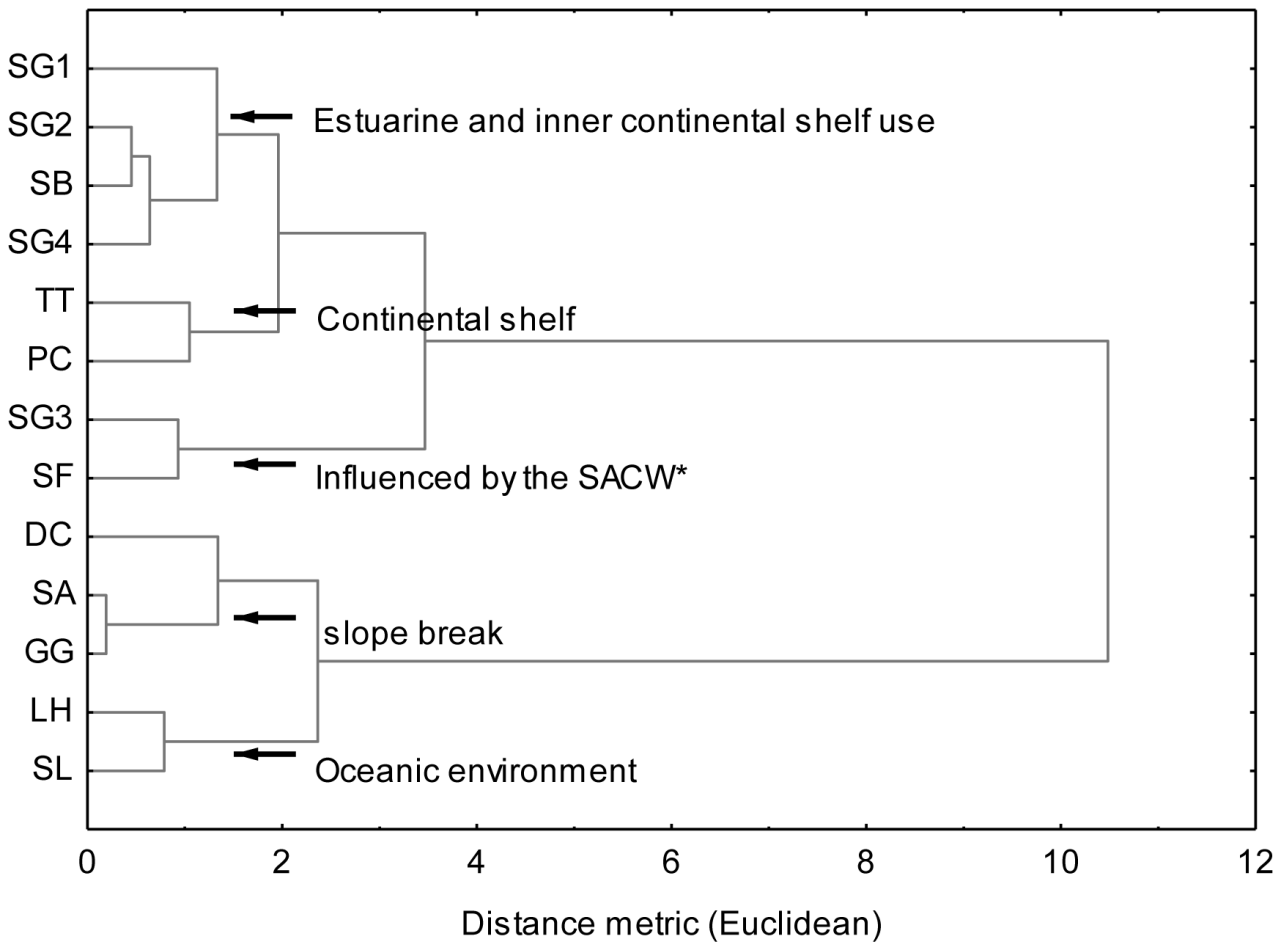
Results of the cluster analysis (Ward's methods) based on δ^13^C and δ^15^N in delphinids muscle tissues. SG1 - *Sotalia guianensis* from Guanabara Bay, SG2 - *S. guianensis* from Sepetiba Bay, SG3 - *S. guianensis* from Ilha Grande Bay, SG4 - *S. guianensis* from “Região dos Lagos”, SB - *Steno bredanensis*, TT - *Tursiops truncatus*, SF - *Stenella frontalis*, PC - *Pseudorca crassidens*, DC - *Delphinus* sp., LH - *Lagenodelphis hosei*, SA - *Stenella attenuata*, SL - *Stenella longirostris* and GG - *Grampus griseus*. *SACW – South Atlantic Central Water.

Using δ^13^C and δ^15^N values, cluster analysis (Ward's method) identified five groups among the delphinid species (Fig. 4). The analysis showed a carbon isotopic continuum, with the highest values in estuarine dolphins (Guiana dolphin), and the lowest values in oceanic delphinids (spinner and Fraser's dolphins). The five groups found were classified as follows: 1) estuarine dolphins and species that use the inner continental shelf (Guiana dolphins from Guanabara Bay, Sepetiba Bay and “Região dos Lagos” and rough-toothed dolphin); 2) continental shelf species (bottlenose dolphin and false killer whale); 3) species influenced by the South Atlantic Central Water (SACW) (Guiana dolphins from Ilha Grande Bay and Atlantic spotted dolphin); 4) shelf-slope species (common, Risso's and pantropical spotted dolphin); and 5) oceanic species (Fraser's and spinner dolphin).

We compared adult males, adult females and calves from Guanabara and Sepetiba bays. There was no significant difference in δ^13^C values in individuals from Guanabara Bay (ANOVA F_(2.22)_ = 0.26; p = 0.77), although there was a difference in δ^15^N values (ANOVA F_(2.22)_ = 6.44; p = 0.006). Calves showed higher δ^15^N values in relation to adult males and females; the adult males and females themselves did not show differences (Unequal N HSD test; p<0.02 and p>0.98, respectively) (Table 3). There was difference between adult males, adult females and calves from Sepetiba Bay both for δ^13^C (ANOVA F_(2.44)_ = 4.93; p = 0.011) and δ^15^N values (ANOVA F_(2.44)_ 34.99; p<0.00001) (Table 3). The *post-hoc* test showed that there was no significant difference between males and females for δ^13^C and δ^15^N values (Unequal N HSD test; p<0.99 and p = 0.08, respectively). Calves had higher δ^15^N values than adults (Unequal N HSD test; p<0.0001). For specimens from Ilha Grande Bay, the only possible comparison performed was between adult males and females due to the absence of calf samples. Values of δ^13^C and δ^15^N were similar for both sexes (Table 3; *t*-test; t = 1.16, p = 0.28 and t = 0.40, p = 0.69, respectively).

**Table 3:** Mean (±SD) δ^13^C and δ^15^N values of muscle tissues from adult males, adult females and calves^1^ of Guiana dolphins.

	δ^13^C (‰)			δ^15^N (‰)		
	male	female	calf	male	female	calf
Guanabara Bay	−13.6±0.6	−13.8±0.6	−13.5±1.1	14.2±1.1	14.4±0.7	16.0±1.3
	(n = 11)	(n = 8)	(n = 6)	(n = 11)	(n = 8)	(n = 6)
Sepetiba Bay	−14.6±0.9	−14.6±1.0	−13.2±0.6	13.9±0.6	14.4±0.4	16.2±0.7
	(n = 29)	(n = 13)	(n = 5)	(n = 29)	(n = 13)	(n = 5)
Ilha Grande Bay	−16.4±0.3	−16.8±0.6	-	14.4±0.6	14.02±0.8	-
	(n = 6)	(n = 3)		(n = 6)	(n = 3)	

[72].^1^ Specimens measuring up to 117.5 cm

## Discussion

Analysis of carbon isotopes has proven to be a very useful tool for identifying differences in both inter-[19], [30] and intra-specific [29], [35] habitat preference. Delphinid δ^13^C values revealed differences between species and allowed us to define groups according to their foraging habitat. There was a significant decrease in δ^13^C values from estuarine dolphins to oceanic species, indicating coast-ocean variation in isotopic ratios. Similar results were observed in other studies involving cetaceans [19], [29], [30], [36]. These differences are due to distinct δ^13^C values in primary sources of carbon in food webs, with coastal and/or benthic systems having higher values than oceanic and/or pelagic systems [24], [26], [27].

Guiana dolphin is a species that inhabits estuarine and coastal regions throughout its distribution [37] and is found in the three coastal bays of Rio de Janeiro State [3], [38], [39]. Among the species studied, Guiana dolphins from Guanabara Bay had the highest average δ^13^C values, even when compared with the same species from Sepetiba and Ilha Grande bays. For dolphins from Guanabara Bay, high site fidelity [3] and predation primarily on demersal, estuarine fish [40] result in the population being under the constant influence of the interior waters of that bay, which may explain the high δ^13^C values.

Site fidelity of Guiana dolphin has also been observed in Sepetiba Bay [38]. However, some authors suggest that Ilha Grande Bay is also used by individuals from Sepetiba Bay [41], because these bays are adjacent and connected by a central channel. Our results showed that Guiana dolphins from Sepetiba and Ilha Grande bays have different foraging habitat, with specimens from Ilha Grande having ^13^C-depleted values. δ^13^C values point to two distinct ecological populations in Sepetiba and Ilha Grande bay. These findings corroborate results from previous studies showing differences in the accumulation of organochlorine compounds [42], in sound emission characteristics [43], and in genetic structure [44] between the two populations. These results from previous studies, in conjunction with the stable isotope data, suggest that movement of Guiana dolphins between the two bays is not frequent, further suggesting that the species shows high site fidelity [3].

The δ^13^C values of Guiana dolphins from Sepetiba Bay varied widely, ranging from −16.9‰ to −12.8‰. This finding may indicate the existence of distinct food sources for this population, suggesting that some individuals forage outside the bay rather than feeding exclusively within Sepetiba Bay. Dias et al. [45] found different distribution patterns between Guiana dolphin “groups” (one to 90 individuals) and “aggregations” (more than 100 individuals) in Sepetiba Bay; most “groups” were observed at the entrance, while most “aggregations” were recorded in the interior of the bay [45]. Further investigations focusing on these groups/aggregations will help to elucidate the existence of distinct foraging/feeding behavior in the Guiana dolphin population from Sepetiba Bay.

Guiana dolphins from Ilha Grande Bay had lower δ^13^C values compared to specimens from the other bays investigated, with values close to those of oceanic delphinids. It is important to highlight that the species is typically a coastal species and, to date, no sighting has been described in the oceanic environment [37]. Bisi et al. [46] also verified that cephalopods and fish with different feeding habits in Ilha Grande Bay were ^13^C-depleted. Furthermore, Ilha Grande Bay is a semi-open system that is more heavily influenced by the colder, more saline water from the marine current flowing from the continental shelf than are Guanabara and Sepetiba bays [47], [48]. Our findings suggest that the low δ^13^C values in Guiana dolphins from Ilha Grande Bay were due to the influence of external water in this estuarine environment.

The four populations of Guiana dolphins had similar δ^15^N values, indicating that they are feeding on prey from the same trophic level. However, it is believed that there may be differences in the trophic position of these populations due to variation in the nitrogen isotopic composition at the base of the food webs among different systems. Bisi et al. [46] suggested that δ^15^N values were reduced at the base of the Guanabara Bay food web. The same authors verified that Guiana dolphins from Guanabara Bay occupy the top trophic level of the food web in this estuary, exhibiting the highest δ^15^N values among the different organisms studied. In contrast, the specimens from Sepetiba Bay are feeding on organisms that occupy relatively lower trophic levels [46]. Thus, although Guiana dolphins from Guanabara Bay showed similar δ^15^N values to those of other populations of the species, they may occupy a higher trophic position in the food web.

Previous studies on marine mammals have shown that feeding ecology may or may not vary between males and females [20], [21], [49], [50]. This study found no influence of sex on feeding of Guiana dolphins in Guanabara, Sepetiba or Ilha Grande bays. Furthermore, calves had higher δ^15^N values than adults in Guanabara and Sepetiba bays. These findings are probably due to isotopic fractionation during the assimilation of breast milk, as calves occupy a higher “trophic level” than their mothers during nursing periods [49]. Our results are in accordance with similar studies conducted on other marine mammal species [20], [49], [50].

Guiana dolphins from Ilha Grande Bay and the Atlantic spotted dolphin were grouped by cluster analysis. Except for two specimens, the Atlantic spotted dolphins sampled were obtained from beaches of “Região dos Lagos”, an area influenced by the South Atlantic Central Water (SACW) upwelling during the summer [51], [52]. SACW also enters Ilha Grande Bay in the summer season, influencing the richness, diversity and abundance of organisms [53]. More negative δ^13^C values of organic carbon dissolved in waters under the influence of SACW have been observed [54], and the similarity of δ^13^C values between Guiana dolphins from Ilha Grande Bay and Atlantic spotted dolphins suggest that SACW influences the foraging areas of these two species along the coast of Rio de Janeiro State.

Rough-toothed dolphins are typically found in oceanic regions [55], but in Brazil they are commonly observed in shallow and coastal waters [55], [56], [57]. The results of the δ^13^C analysis suggest that the species uses continental shelf waters in southeastern Brazil, primarily foraging along the inner part of the shelf. This hypothesis is reinforced by the results of the cluster analysis, in which Guiana and rough-toothed dolphins shared the same group.

Bottlenose dolphins and false killer whales occupied similar trophic niches, with similarities in δ^13^C and δ^15^N values. These species had the highest δ^15^N values, suggesting that these animals occupy the highest trophic level among the delphinids considered in this study. Stomach content analyses showed that bottlenose dolphins feed mainly on teleost fish and cephalopods along the south-central coast of Rio de Janeiro State [14]. Moreover, fish preyed upon by this species were significantly larger than those preyed upon by other delphinids in this region. False killer whales also feed on fish and cephalopods, but the intake of small cetaceans has also been reported [56], [58]. In addition, some studies have shown distinct foraging patterns for false killer whales in the South Atlantic Ocean based on δ^15^N values 19,36. The false killer whale with low δ^15^N values are probably feeding specifically on cephalopods, whereas individuals that are ^15^N-enriched would be feeding at higher trophic levels (e.g., fish) [19], [36]. Due to the high δ^15^N values found in this study, it is likely that false killer whales prey mainly on high-trophic-level fish or even on marine mammals. These results are in accordance with the high concentrations of organohalogen compounds found in tissues of false killer whales from the study region, which suggest regular feeding on marine mammals [59], [60]. Bottlenose dolphins and false killer whales had similar δ^13^C values to other nearshore species (rough-toothed and Guiana dolphins), suggesting that these species also forage in the region along the continental shelf, with similar habitat preferences. This is a relevant finding because studies have reported the use of oceanic habitats, in waters of greater than 1,000 m, for false killer whales [56], [58] and have revealed a distinct foraging pattern throughout the species distribution [19], [36], [56]. However, the results of this study highlight the limited knowledge about that habitat preferences of this species.

The δ^13^C values indicated that Risso's and pantropical spotted dolphins inhabit waters along the continental shelf break. A similar result was observed for Risso's dolphins in Tierra del Fuego, Argentina [19], as well as those found along the northwest coast of Africa [30]. Spinner and Fraser's dolphins had the lowest δ^13^C values, lending further support to the described use of oceanic habit [56]. These oceanic species also had the lowest δ^15^N values and were found in groups four (Risso's dolphin and pantropical spotted dolphin) and five (spinner dolphin and Fraser's dolphin), identified from the cluster analysis. Studies have shown a positive correlation between trophic level and δ^15^N values [31], [32], [61]. Nevertheless, δ^15^N values of the isotopic baseline can vary considerably among ecosystems and regions [24], [30], [62]. Thereby, our δ^15^N results may reflect oceanic species feeding on low trophic level prey or could be due to the low δ^15^N values at the base of the ocean food web. An important source of nitrogen in the ocean's photic zone is in the form of nitrate, which typically features higher δ^15^N values of approximately 6 ‰ [63], [64]. On the other hand, several studies have associated low δ^15^N values in the biota to the influence of atmospheric N_2_ fixation by cyanobacteria in oceanic waters [65], [66], [67], which seems to be a much greater source of nitrogen than assumed in the past [65]. The low δ^15^N values in oceanic dolphin species point to a substantial input of N_2_ fixed by cyanobacteria rather than nitrate as a primary source of nitrogen in foraging areas.

Among the oceanic species, pantropical spotted and Risso's dolphins had the lowest δ^15^N values. Stomach content analyses have shown that pantropical spotted dolphins feed mainly on mesopelagic fish of the Myctophidae family, as well as on cephalopods from the families Enoploteuthidae and Ommastrephidae [13], [68]. Risso's dolphins feed almost exclusively on cephalopods, primarily from the families Octopodidae, Loliginidae and Ommastrephidae [69], [70], [71]. These studies showed that these two species had some similar prey types, such as ommastrephid squids. In the present study, δ^13^C and δ^15^N values were very similar among pantropical spotted and Risso's dolphins, suggesting a large overlap in foraging area or prey consumed.

## Conclusions

This study provides new information on the trophic ecology of 10 delphinid species, including four populations of Guiana dolphins, in southeastern Brazil. Evidence from δ^13^C and δ^15^N values indicated that there was segregation among the delphinids occurring along the coast of Rio de Janeiro State, with species having distinctive foraging habitats and occupying different ecological niches. For example, rough-toothed dolphins appear to forage along the inner shelf, whereas bottlenose dolphins and false killer whales use the continental shelf. Values of δ^13^C suggest that Risso's and pantropical spotted dolphins forage along the platform break, while spinner and Fraser's dolphins used similar oceanic habitat. Bottlenose dolphins and false killer whales occupied the highest trophic position, while spinner and Fraser's dolphins fed on lower trophic level prey. However, investigations regarding the δ^15^N values at the base of food webs in different environments are necessary for a better understanding of the trophic levels occupied by delphinid species. Lastly, δ^13^C values showed a clear separation between the Guiana dolphin populations from adjacent areas. It is important to emphasize that the delphinid species studied occur in a region under high anthropogenic pressure, subject to pollution, intense vessel traffic, oil exploration, seismic prospecting, and intense commercial fishing, among other factors. Knowledge and understanding of the habitat preferences of delphinids in southeastern Brazil is of fundamental importance for identifying potential threats to which these animals are subjected, as well as for supporting appropriate conservation actions.
